# Two Estrogen Response Element Sequences Near the *PCNA* Gene Are Not Responsible for Its Estrogen-Enhanced Expression in MCF7 Cells

**DOI:** 10.1371/journal.pone.0003523

**Published:** 2008-10-24

**Authors:** Cheng Wang, Jie Yu, Caleb B. Kallen

**Affiliations:** Department of Gynecology and Obstetrics, Emory University School of Medicine, Atlanta, Georgia, United States of America; Institute of Genetics and Molecular and Cellular Biology, France

## Abstract

**Background:**

The proliferating cell nuclear antigen (PCNA) is an essential component of DNA replication, cell cycle regulation, and epigenetic inheritance. High expression of PCNA is associated with poor prognosis in patients with breast cancer. The 5′-region of the *PCNA* gene contains two computationally-detected estrogen response element (ERE) sequences, one of which is evolutionarily conserved. Both of these sequences are of undocumented *cis*-regulatory function. We recently demonstrated that estradiol (E2) enhances *PCNA* mRNA expression in MCF7 breast cancer cells. MCF7 cells proliferate in response to E2.

**Methodology/Principal Findings:**

Here, we demonstrate that E2 rapidly enhanced *PCNA* mRNA and protein expression in a process that requires ERα as well as *de novo* protein synthesis. One of the two upstream ERE sequences was specifically bound by ERα-containing protein complexes, *in vitro*, in gel shift analysis. Yet, each ERE sequence, when cloned as a single copy, or when engineered as two tandem copies of the ERE-containing sequence, was not capable of activating a luciferase reporter construct in response to E2. In MCF7 cells, neither ERE-containing genomic region demonstrated E2-dependent recruitment of ERα by sensitive ChIP-PCR assays.

**Conclusion/Significance:**

We conclude that E2 enhances *PCNA* gene expression by an indirect process and that computational detection of EREs, even when evolutionarily conserved and when near E2-responsive genes, requires biochemical validation.

## Introduction

The proliferating cell nuclear antigen (*PCNA*) gene product is a nuclear protein that acts as a cofactor for DNA polymerase-δ and participates in DNA synthesis [1] and repair [2](for reviews see [3], [4]). In addition, by interacting with a wide array of proteins, PCNA serves essential functions in cell cycle progression [5], epigenetic inheritance [6], [7], and gene transcription [8], [9]. *PCNA* gene expression is generally low in quiescent cells, increases with cell proliferation [10], and is tightly controlled within the cell cycle. In response to proliferative stimuli, *PCNA* mRNA and protein levels both increase during the G1/S transition, commensurate the protein's role in DNA replication [11]–[14].

PCNA synthesis is induced by diverse stimuli in a cell-type specific fashion, including: EGF, PDGF, and serum in 3T3 cells [15], [16], interleukin 2 (IL-2) in T-lymphocytes [17], and p53 [18] and adenovirus infection in HeLa cells [19]. There appear to be transcriptional and post-transcriptional mechanisms for regulating *PCNA* mRNA levels in 3T3 cells by processes that are not fully characterized [10], [17], [20], [21]. No formal study of *PCNA* gene regulation has been demonstrated in breast cancer cells.

Most studies have observed that high *PCNA* gene expression correlates with increased metastatic potential and decreased survival in patients with breast carcinoma [22]–[28]. Many breast and uterine cancers depend upon E2 for neoplastic initiation, development, or metastasis, and antiestrogen therapies remain the mainstay of treatment and prevention for ERα-expressing breast cancers. The E2 response in breast cancer cells is predominantly mediated by ERα, a ligand-activated transcription factor [29].

We confirmed that *PCNA* gene expression is enhanced by E2 exposure in MCF7 breast cancer cells which express ERα and proliferate in response to E2 [30], [31]. We, and others, have detected two putative estrogen response elements (EREs) in the 5′ region of the *PCNA* gene, one of which is conserved between murine and human species, and both of which may serve as *cis*-regulatory elements for ERα-mediated gene regulation [32]. Recently, PCNA was shown to physically interact with ERα [33] and RARα [34] and to modulate gene transcription regulated by these transcription factors. These observations raise the possibility that E2-stimulated ERα activates *PCNA* gene expression, leading to feedback regulation of ERα transcriptional functions by ERα-bound PCNA. The process of *PCNA* gene induction is likely to be essential to the mitogenic effects of E2 in some ERα-expressing cancers.

The *PCNA* promoter is regulated at the transcriptional level by many transcription factors including E1A [35], [36], ATF1 [37], RFX1 [38], CBP [39], p107 [40], p53 [18], [19], [41], and E2F [11], [12]. In some systems, basal transcription is augmented at G1/S by inducible regulatory elements [12]. No role for ERα has been demonstrated in the regulation of *PCNA* gene expression although estrogens act as potent mitogens in both normal and neoplastic breast and uterine tissues. Because eukaryotic *cis*-regulatory elements may reside great genomic distances from target genes [31], [42]–[45], and because the putative EREs that we identified are located 1,200–10,000 bp from either transcription start site (TSS) demonstrated for *PCNA*, we thought it important to test these elements for functional significance. Our goals were to understand the predictive value of computational ERE detection for an E2-responsive gene and to better define the mechanisms by which estrogen stimulates *PCNA* gene expression in breast cancer cells. Our data indicate that E2 enhances *PCNA* gene expression by an indirect process and that computational detection of EREs, even when evolutionarily conserved and when near E2-responsive genes, requires biochemical validation.

## Results

### E2 stimulated *PCNA* mRNA and protein expression in a process that requires *de novo* protein synthesis

We recently reported the results of microarray-based gene expression profiling using the MCF7 breast cancer cell line, a model system for E2-dependent breast tumors [31]. MCF7 cells express ERα and proliferate in response to E2 exposure. We observed increased *PCNA* gene expression after 4, 12, and 24 hours of E2 exposure. Notably, two putative EREs were previously detected upstream of *PCNA* by Bourdeau *et al*, who applied large scale computational analyses to the human and mouse genomes for detection of conserved ERE sequences [32]. Our analysis revealed that the both ERE sequences are 100% conserved between Rhesus and human, whereas the 3′-ERE sequence also shares 79% identity with mouse, indicating that the 3′-ERE is more evolutionarily conserved. These ERE sequences were never tested for function.

**Figure 1:**
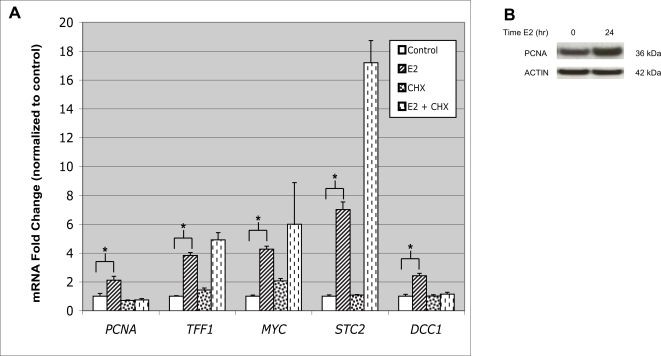
Estrogen stimulates *PCNA* mRNA and protein expression in MCF7 cells. (A) MCF7 cells were treated for six hours with or without E2 (100 nM) and with or without CHX (25 µg/ml), an inhibitor of protein synthesis. RNA was collected and subjected to Q-RT-PCR. Known E2-responsive genes included *TFF1, MYC, STC2*, and *DCC1*. The E2-stimulated expression of *PCNA* and *DCC1* was lost when cells were co-treated CHX. Values are the average of three experiments, performed in triplicate, with SEM indicated. (B) PCNA protein levels were measured from MCF7 cell lysates by Western blot after 0, and 24 hours of E2 (10 nM) exposure. Results using ACTIN-specific antibody on the same lysates are also shown. The data are representative of an experiment performed twice. * P < 0.02 comparing control with E2-treated.

Quantitative reverse transcription polymerase chain reaction (Q-RT-PCR) was applied to MCF7 cell lysates and confirmed greater than 2-fold induction of *PCNA* mRNA after six hours E2 exposure (Figure 1A). Known E2-responsive genes also tested include *TFF1, MYC, STC2*, and *DCC1.* Similar changes in PCNA protein levels were observed after E2 treatment of MCF7 cells (Figure 1B). The E2-stimulated expression of *PCNA* mRNA was sensitive to co-treatment with the protein synthesis inhibitor cycloheximide (CHX), suggesting a secondary, or indirect, transcriptional effect of E2 exposure (Figure 1A). Interestingly, DCC1, a component of the replication factor C (RFC) which loads PCNA onto DNA during DNA replication, demonstrated expression that was similarly E2 responsive and CHX sensitive. These data are consistent with a model in which DNA replication is regulated within the cell cycle, in part, by the regulated synthesis and degradation of the replicative machinery [46].

There exist direct transcriptional targets of ERα that require *de novo* protein synthesis in order to be transcriptionally regulated by the receptor. For example, the ERα gene target *c-fos* is rapidly activated by E2 in uterine tissues [47], [48] whereas *de novo* protein synthesis is required in order to produce a sustained *c-fos* transcriptional response to E2 in MCF7 cells [49]. Similarly, there exist gene targets that are repressed by ERα only after induction of the corepressor protein NRIP-1 [44]. Thus, the fact that the *PCNA* response to E2 was CHX-sensitive did not preclude the gene from being a direct responder to ERα.

### E2-enhanced *PCNA* gene expression was blocked by inhibition of ERα function

**Figure 2:**
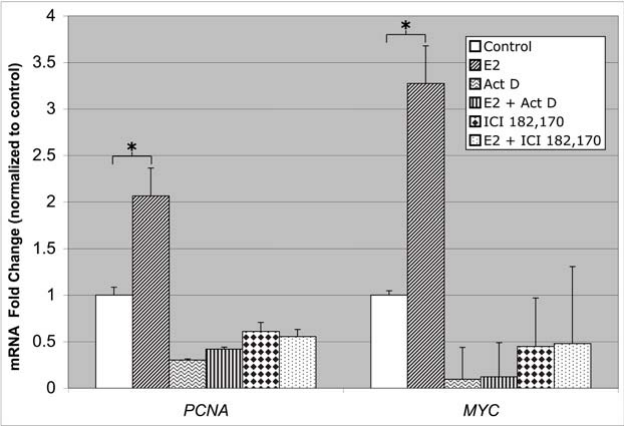
Estrogen-stimulated *PCNA* gene expression requires ERα and *de novo* gene transcription. MCF7 cells were treated with or without E2 (100 nM) for 6 hours. Additional treatments included ActD (2 µg/ml), an inhibitor of gene transcription, and ICI 182,780 (1 µM), a pure estrogen receptor antagonist. RNA was collected and subjected to Q-RT-PCR. *PCNA* mRNA levels were compared with *MYC* mRNA levels from the same samples. Values are the average of three experiments, performed in triplicate, with SEM indicated. * P < 0.05 comparing control with E2-treated.

Blocking an E2-mediated transcriptional response by co-treatment with ICI 182,780, a pure ERα antagonist, indicates that the observed effect is mediated by ERα [50], [51]. In MCF7 cells, the E2-stimulated expression of *PCNA* was blocked by co-treatment with ICI 182,170 and by inhibition of gene transcription using actinomycin D (ActD) (Figure 2). Similar results were observed for *MYC* gene expression, which is also E2-responsive and a direct gene target of ERα [52]. These data support the hypothesis that E2-stimulated *PCNA* gene expression requires both the activity of ERα and *de novo* gene transcription.

**Figure 3:**
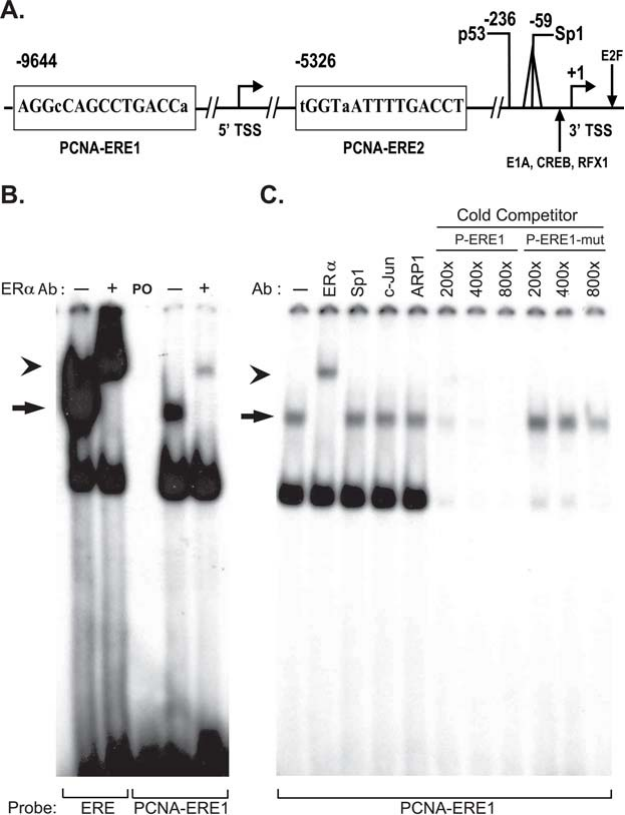
Two EREs reside near the TSSs for *PCNA*, one binds to ERα *in vitro.* (A) Genomic sequences of two EREs, the 5′-ERE (PCNA-ERE1) and the 3′-ERE (PCNA-ERE2), are indicated, with associated distances from the 3′ transcription start site (TSS) of the *PCNA* gene. Deviations from the consensus ERE sequence are indicated in lower case. Previously-described regulatory regions are similarly indicated (see main text). (B) EMSA was performed using HESC cell nuclear lysates plus recombinant ERα (rERα) and radiolabeled consensus ERE or PCNA-ERE1 probes. The ERα-containing complex (arrow) is demonstrated by supershift (arrowhead) using a monoclonal antibody that recognizes ERα. In the middle lane, “PO” indicates probe only, and lacks proteins. (C) EMSA data as in (B), wherein supershift is noted using antibody specific for ERα but not when using antibodies for ERα tethering partners Sp1, c-Jun (a member of AP1 complexes), or ARP1/COUP-TFII. ERα-containing radioactive complexes are efficiently competed away by unlabeled (“cold”) wild-type PCNA-ERE1 sequences (P-ERE1) but not with cold probe sequences mutated at the ERE-containing residues (P-ERE1-mut).

We recently demonstrated that siRNA-mediated knockdown of *PCNA* gene expression greatly inhibited E2-stimulated cell proliferation in MCF7 cells [31]. These data supported the hypothesis that PCNA is an important mediator of E2-stimulated cell proliferation in MCF7 cells. Our analysis of the *PCNA* gene locus confirmed two imperfect ERE's within 10 Kb of the two TSSs described for the gene (Figure 3A) [12], [17]. Each putative ERE (herein dubbed PCNA-ERE1 and PCNA-ERE2) demonstrates a single nucleotide mismatch from the core 13 bp consensus (or “canonical”) ERE sequence. Notably, the majority of EREs identified and validated in the human genome do not demonstrate perfect consensus sequences, indicating a high degree of heterogeneity for functional ERE sequences [53]–[57]. Similarly, the mere presence of an ERE-like sequence is not sufficient to ensure ERα binding or ERα-mediated transcriptional responses in the majority of chromatin contexts [31], [58]. These observations indicate that ERE sequences must combine with additional factors in order to function. Such factors may include regional histone composition, distribution, and post-translational histone modifications, DNA methylation status, and regional DNA sequences (with associated *trans*-factors) that create a transcriptionally permissive environment for activated ERα [59].

### A predicted ERE sequence near the *PCNA* gene was capable of binding to ERα *in vitro*

We performed electrophoretic mobility shift assays (EMSA) using the radiolabeled PCNA-ERE1 sequence with recombinant ERα protein. In order to promote receptor-DNA binding, and to control for the presence or absence of ERα protein, recombinant ERα was combined with cofactors present in nuclear extracts from an ERα-negative, immortalized human endometrial stromal cell (HESC) line [60]. ERα-containing protein complexes were shown to bind to PCNA-ERE1, *in vitro*, as evidenced by supershift with an antibody specific for ERα (Figure 3B). No estrogen receptor-containing complex was noted from HESC cell extracts alone (not shown). No supershift was noted using antibodies targeting the transcription factors Sp1, AP-1 or ARP-1/COUP-TF2, although these factors have been demonstrated to bind with ERα at selected promoters (Figure 3C) [61]–[63]. Binding was weaker for PCNA-ERE1 than that observed for a consensus ERE sequence (Figure 3B) but stronger than that observed with PCNA-ERE2, for which binding was weakly detectable (not shown).

ERα-dependent binding to radiolabeled PCNA-ERE1 could be competed away using excess unlabeled (“cold”) PCNA-ERE1 probe (P-ERE1) whereas a similar probe with mutations in the two half-sites of the ERE (P-ERE1mut) did not efficiently compete for labeled PCNA-ERE1-bound receptor (Figure 3C). These data suggested that one or both of these putative EREs might represent ERα-responsive *cis*-regulatory elements that modulate *PCNA* gene expression in MCF7 cells. It is recognized that the affinity of receptor binding to an ERE, as measured *in vitro*, need not correlate with the potency of the enhancer function that is observed [53] and that multiple EREs can work cooperatively in order to enhance target gene transcription [64], [65].

### Two ERE sequences near the *PCNA* gene did not enhance reporter expression in response to E2 treatment

**Figure 4:**
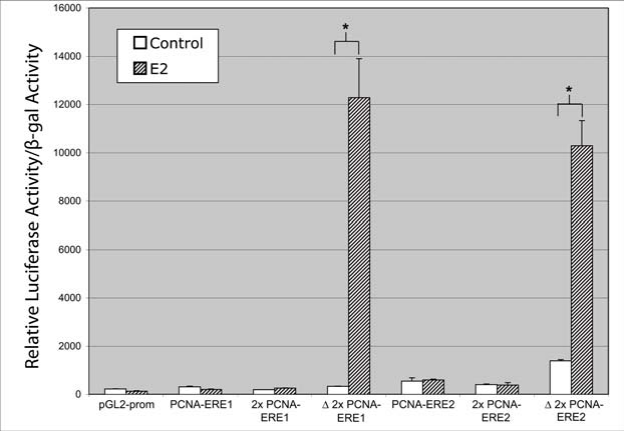
Two EREs near *PCNA* do not enhance expression of a reporter in response to estrogen. The 822 bp genomic fragment containing PCNA-ERE1 and the 551 bp genomic fragment containing PCNA-ERE2 do not enhance luciferase reporter activity in response to E2. Two tandem copies of the isolated 15 bp ERE sequence found within PCNA-ERE1 (2×PCNA-ERE1) or PCNA-ERE2 (2×PCNA-ERE2) did not enhance reporter activity in response to E2. When these tandem ERE sequences were mutated to produce perfect 13 bp core consensus ERE sequences (Δ-2×PCNA-ERE1 and Δ-2×PCNA-ERE2) they produced E2-dependent enhancement of reporter activity in MCF7 cells. Values are the average of three experiments, performed in triplicate, with SEM indicated. * P < 0.002 comparing E2-treated with control.

We cloned genomic fragments corresponding to each PCNA-ERE into luciferase reporter constructs and tested these for enhancer function, *in vitro*. Surprisingly, neither an 822 bp fragment containing PCNA-ERE1 nor a 551 bp fragment containing PCNA-ERE2 demonstrated E2-inducible enhancer function in MCF7 cells (Figure 4). Similar results were obtained when these constructs were tested with co-expressed ERα in HESC cells (which do not express endogenous ERα but respond to E2 when made to express ERα (not shown)). Thus, changing the cell type in which we tested the reporter constructs, and presumably the cohort of available transcription factor co-regulatory proteins within the cell nucleus [66], failed to indicate enhancer function for the putative ERE sequences being tested.

Luciferase reporter assays that use single copy response elements are sometimes weakly responsive to transcription factors. Further, the function of any cloned enhancer region may be subject to inhibition by neighboring *cis*- and *trans*-regulatory elements, depending upon the length of DNA that is cloned and the inclusion or exclusion of such regulatory elements in the reporter construct. This phenomenon could produce false-negative observations in luciferase reporter assays that would depend, in part, upon the size of the genomic fragment that is employed in the assay. It is, therefore, common to assay multiple tandem copies of *cis*-regulatory elements in order to demonstrate enhancer function, *in vitro*.

We engineered luciferase reporter constructs with two tandem copies of the 15 bp PCNA-ERE1 sequence or two copies of the PCNA-ERE2 sequence (2×PCNA-ERE1 and 2×PCNA-ERE2), to test these isolated sequences for enhancer activity. As seen with the larger ERE-containing genomic fragments, the 2× tandem ERE sequences did not demonstrate E2-responsive enhancer function in MCF7 cells (Figure 4). When each tandem ERE sequence was mutated by one nucleotide to conform to a perfect 13 bp consensus ERE (Δ-2×PCNA-ERE1 and Δ-2×PCNA-ERE2), strong E2-responsive enhancer function was observed (Figure 4). These results confirmed that the promoter-reporter construct, pGL2-promoter, is functional in response to E2 in MCF7 cells when harboring *bona fide* ERE enhancer sequences. Taken together, these data did not support the hypothesis that the putative EREs, each computationally detected, are likely to function as ERα-regulated enhancer elements for the *PCNA* gene in MCF7 cells.

### Two ERE sequences near the *PCNA* gene were not bound by ERα *in vivo*

In light of the fact that the ERE-like sequences near *PCNA* are nearly consensus EREs, and that *PCNA* gene expression is E2-responsive, we wondered whether the *in vitro* assays that we employed to detect enhancer function could have produced spurious results. It remained possible that the chromatin context, *in vivo,* might dictate enhancer function in ways not observed using plasmid DNA in reporter assays. Imperfect (i.e. non-consensus) EREs have been demonstrated to have function when optimally positioned with regards to target TSSs, wherein they can provide sufficient affinity for ERα binding, permit important DNA bending, and favor specific patterns of coregulator recruitment to the target gene promoter [67]–[69].

Although we were unable to detect ERα binding to PCNA-ERE1 or PCNA-ERE2 using ChIP-on-chip in response to E2 [31], we reasoned that this result could reflect established limitations in the sensitivity of this microarray-based approach. When we compared our findings to other reports of genome-wide location analysis for ERα in MCF7 cells, we similarly found no evidence for recruitment of ERα to the PCNA gene locus using ChIP-on-chip or alternative genomic approaches such as ChIP-DSL and ChIP-PET [44], [70], [71]. Notably, the receptor location analysis work in these studies compared ERα target occupancy after chromatin immunoprecipitation using E2-treated cells relative to ERα occupancy of chromatin DNA prepared from cells not subjected to immunoprecipitation (i.e. sheared input). As such, the approaches were not optimally designed to detect changes in target occupancy that depend upon E2 exposure, which can be tested by comparing ChIP of untreated cells (vehicle-treated ChIP) with ChIP from E2-treated cells.

**Figure 5:**
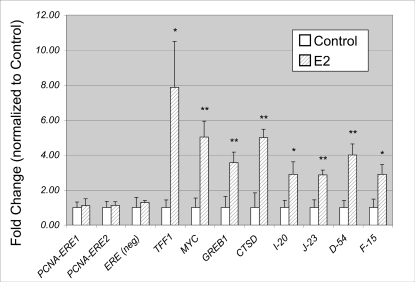
ERα is not recruited to genomic PCNA-ERE1 or PCNA-ERE2, *in vivo,* after E2 exposure. MCF-7 cells were treated with or without E2 for 45 minutes, then ChIP was performed using antibodies against ERα. Quantitative PCR using genomic primers residing within 500 bp of the putative ERE sequences revealed no evidence that ERα is recruited to either putative ERE in response to E2 exposure. E2-stimulated ERα similarly failed to be enriched at an ERE-less locus (ERE-neg) that was also negative in prior ChIP-on-chip studies. Similar studies using genomic PCR primers for the E2-responsive genes *TFF1*, *MYC*, *GREB1*, and *CTSD* demonstrated rapid recruitment of ERα to these enhancer regions in response to E2. Also shown is ChIP-PCR validation of ERα-bound loci detected using ChIP-on-chip, as previously reported [31], targeting loci I20, J23, D54, and F15 (see methods section). Values are the average of three experiments, with SEM indicated. * P < 0.1 comparing E2-treated with control. ** P < 0.05 comparing E2-treated with control.

In order to test for E2-dependent binding of ERα to PCNA-ERE1 or PCNA-ERE2, we performed ChIP-PCR using primers that span each putative ERE. Using ChIP-PCR, we were unable to demonstrate recruitment of ERα to either putative ERE in response to E2 (Figure 5). Similar results were obtained using multiple alternative PCR primer pairs targeting the same genomic regions (not shown). *In toto*, more than 3 primer pairs were used to evaluate ERα occupancy at each putative *cis*-element in response to E2; all demonstrated no evidence for receptor recruitment to the ERE sequences in response to hormone. The ChIP-PCR data shown in Figure 5 included a 45 min exposure to E2, a treatment that has been demonstrated to produce optimal ERα recruitment to target enhancers [72]. We obtained identical results with E2 treatments extended for 3 and 6 hours prior to protein crosslinking (not shown).

A genomic locus (chr7:72384008-72385027) that lacks an ERE and that failed to be detected using ChIP-on-chip (dubbed ERE-neg) also failed to be enriched for ERα using ChIP-PCR when comparing control and E2-treated cells (Figure 5). By contrast, ChIP-PCR confirmed E2-dependent recruitment of ERα to several known genomic targets including *TFF1*, *MYC*, *GREB1*, and *CTSD.* In addition, ChIP-PCR confirmed E2-dependent recruitment of ERα to four targets that we previously identified using ChIP-on-chip [31], dubbed I20, J23, D54, and F15 (see methods for genomic locations) (Figure 5). These data suggest that neither of the putative EREs detected near *PCNA* binds to ERα in an E2-dependent fashion, *in vivo*, in MCF7 cells. Further, the data demonstrate a good correlation between our ChIP-on-chip datasets and ChIP-PCR datasets.

## Discussion

Our data show that PCNA, an essential participant in DNA replication, epigenetic programming, and a regulator of the cell cycle, is up-regulated by E2 exposure in MCF7 cells. Epigenetic alterations play critical roles in tumorigenesis and cancer progression [73], [74] and we recently demonstrated that high protein expression of the E2-responsive histone variant H2A.Z in primary breast tumors correlates with decreased patient survival [31]. It is attractive to consider, and previously has been postulated [30], [33], that the E2-dependent expression of *PCNA* is potentially important for the proliferation of a diversity of tissues and tumors. Nair and colleagues recently reported an ERα-dependent proliferative effect in which estrogen enhanced *PCNA* gene expression in cancers of the cervix but not in normal cervix [75]. E2 exposure is a well-recognized risk factor for cancer of the breast and endometrium, and E2 enhances *PCNA* gene expression in human myometrial and leiomyoma tissues as well [76]. Most-recently, E2-responsive tumor progression has been suggested for epithelial ovarian cancer [77].

Our data indicate that, in MCF7 cells, the E2 effect depends upon new gene transcription and translation and is blocked by the pure ERα-antagonist ICI 182,780. Although excellent computational analysis [32], and preliminary *in vitro* data (EMSA), suggested that the mechanism of *PCNA* gene regulation might include the function of ERE sequences in the 5′ region of the gene, our testing of the most-likely sequence elements, using several approaches, failed to confirm *cis*-regulatory function in MCF7 cells.

That a subset of ERα target genes may require the synthesis of additional cofactor(s) prior to becoming subject to receptor-mediated transcriptional regulation remained a possibility consistent with our preliminary gene expression data. Such a model has been observed for ERα gene targets that are repressed by ERα only after the induction of corepressor protein NRIP-1 [44]. Similarly, some genes targeted by ERα require the function of a chromatin modifier, FOXA1, prior to becoming subject to ERα-mediated transactivation [59], [78]. The observation that E2 engenders gene regulatory cascades that can be divided into temporal categories (i.e. immediate, early, and late responses) is well-demonstrated [30], [31]. These data indicate that some genes are poised for immediate regulation, others may represent downstream/indirect targets of ERα function, and still other gene targets may require the synthesis of cofactors to modify chromatin targets in preparation for the arrival of activated ERα [78]. Neither the timing of a transcriptional response (early vs. late) nor the sensitivity of the response to CHX can be taken as formal proof that a given response is direct (i.e. ERα-mediated) or indirect.

This report describes two ERE-like sequences upstream of an E2-responsive gene. Both sequences failed to demonstrate transcriptional regulatory function *in vitro* and *in vivo* in MCF7 cells. PCNA-ERE1 resides within a repetitive element (Alu-Sc) while PCNA-ERE2 does not. A single report described a functional Alu-ERE for the BRCA1 gene [79] which, on further inspection, was determined to be non-functional in MCF7 cells [80]. The reported ERE sequence in those studies is not the same as noted for PCNA-ERE1. Our results indicate that *PCNA* is regulated in response to E2 either indirectly, or via a *cis*-acting ERE not detected by several independent genome-wide location analyses for ERα [31], [44], [70], [71], and only after the synthesis of newly translated protein(s). Importantly, in addition to ChIP-on-chip approaches, two genome-wide location analyses utilized ChIP-PET and the sensitive ChIP-DSL approaches; all demonstrated no evidence for ERα recruitment to *PCNA*.

Five members of the E2F family of transcription factors (of which humans have at least eight) are up-regulated by E2 in MCF7 cells although direct transcriptional regulation by ERα remains to be established for these genes [31]. Recent data suggests that E2F family members are capable of binding to identical sequences as homodimers or heterodimers (with DP family members) and may often subserve redundant functions [81]. Increasing data indicate that these factors play important roles in the E2-dependent cellular proliferation of breast cancer cells [82], [83]. E2F1 regulates *PCNA* gene expression in some systems [84]. We tested E2F1 using ChIP-PCR and found no evidence for E2-dependent recruitment of E2F1 to the transcription start sites of the *PCNA* gene (data not shown). Similar negative results were obtained when performing ChIP-PCR using antibodies for Sp1 or AP-1 in order to interrogate both *PCNA* ERE-like sequences and the two TSSs of the *PCNA* gene (not shown). ATF1 [37] and CREB1 are also regulators of *PCNA* gene expression but were not enriched at the *PCNA* promoter in MCF7 cells in genome-wide ChIP-chip analyses (S. Hua and K. White, manuscript in preparation).

In order to identify alternative candidates that might mediate the estrogen response of *PCNA* in MCF7 cells, we undertook a computational analysis of predicted transcription factor binding sites in the two promoter regions for *PCNA.* These data were then correlated with gene expression data from our work, and from the work of others, cataloging estrogen-responsive transcription factors in MCF7 cells. The intersection of these datasets provides a list of estrogen-responsive transcription factors with high-confidence binding sites residing within two kilobases of each transcription start site for the *PCNA* gene (Table S2). In addition to E2F family members that warrant investigation (above), we have identified c/EBPβ, FOXC1, FOXJ2, GATA-3, POU2F1/AP-2γ, RARA, TFAP2C, and TFE3 as estrogen-responsive transcription factors with predicted binding sites in one or both promoter regions of the *PCNA* gene. Taken together, these data reveal good candidates for the mediator of the estrogenic cascade leading to enhanced *PCNA* gene expression in MCF7 cells. These candidates will be pursued in our future studies.

Notably, ∼2310 perfect 13 bp consensus EREs (GGTCAnnnTGACC) exist within the human genome. Permitting just one nucleotide mismatch from the consensus sequence reveals nearly 50,000 ERE-like sites throughout the human genome. Our ChIP-on-chip data, supported by biological plausibility, suggest that the overwhelming majority of these sites are not functional in any given cell type. Two groups have used MCF7 cells to perform whole genome ChIP-on-chip for ERα location analysis, revealing between ∼1600–3700 receptor-bound loci in response to E2 (with considerable reproducibility) [31], [44]. Analysis of the highest-confidence ChIP sites, the 1017 sites that are common to both datasets, indicates that more than 90% of the ERE-like sequences at these loci are not perfect consensus sequences. Further, these datasets indicate that, most likely, <5–10% of perfect consensus ERE sites in the genome are ERα-bound in response to E2 in MCF7 cells.

The chromatin and cellular determinants of ERα binding to enhancer elements remain to be fully established. This issue raises a cautionary note when drawing conclusions based upon computational analyses of genomic sequence which, even when evolutionarily conserved, will present hypotheses that must be validated by formal molecular biological testing. We conclude that computational detection of *cis*-regulatory elements in the human genome, even when accompanied by appropriate gene expression data, cannot be taken as proof of *cis*-regulatory element function *in vivo* [32], [85]–[87]. Each element must be tested, preferably using *in vivo* assays such as ChIP-PCR and chromatin conformation capture, in order to confirm *cis*-element function in any given cell type and cell context.

## Materials and Methods

### Cell Culture

MCF7 cells (ATCC) were grown as described [78]. Cells were changed to estrogen-depleted, phenol-free media consisting of MEM alpha (Gibco) with 10% charcoal/dextran-stripped calf serum, insulin (4 µg/ml, Sigma), penicillin G, streptomycin, and L-glutamine (all Gibco), for 72 hours prior to treatments. Where indicated, treatments included vehicle control (100% EtOH), estradiol (10 nM or 100 nM, Sigma), actinomycin D (ActD, 2 µg/mL, Sigma), cycloheximide (CHX, 25 µg/mL, Sigma), and ICI 182,780 (1 µM, Tocris Biosciences). Telomerase-immortalized Human Endometrial Stromal Cells (HESC cells), a generous gift from Dr. Graciela Krikun, were grown in the same media used for the MCF7 cells. HESC cells have normal chromosome numbers and structures [60].

### Preparation of Nuclear Extracts and EMSA

HESC nuclear extracts (NE) were purified using NE-PER Nuclear and Cytoplasmic Extraction Reagents (Pierce) according to the manufacturer's instructions. HESC cells have no demonstrable ERα activity using sensitive luciferase reporter assays and no ERα protein detected by Western blot analysis (data not shown). However, HESC cell nuclei have cofactors that promote the binding of recombinant ERα to target DNA in EMSA and these factors enhance binding when compared to recombinant ERα alone (rERα, Affinity Bioreagents). EMSA experiments were therefore conducted using HESC nuclear extracts combined with rERα. Protein determinations were performed using the Micro BCA assay (Pierce) and 5 µg of nuclear extract (with protease inhibitors, Roche) plus rERα (170 nM) was used in each lane of a 5% acrylamide gel in TBE buffer. Oligonucleotide probes were ^32^P-labeled using T4 Polynucleotide Kinase 5′ End Label System (Promega) and purified using Mini Quick Spin Oligo Columns (Roche). Each radiolabeled probe was used at ∼200,000 cpm/lane and binding reactions included Tris-HCl pH 8.0 (25mM), KCl (50 mM), MgCl2 (6.25 mM), Glycerol (10%), DTT (0.5 mM) plus relevant antibody where indicated (400 ng/reaction): anti-ERα Ab-10 (LabVision), anti-cJun(N) sc-45 (Santa Cruz), anti Sp1 H-225 sc-14027 (Santa Cruz) and anti-ARP1/COUP-TFII (Santa Cruz). A complete list of oligonucleotide sequences used as probes for EMSA is presented in the supplementary materials (Table S1).

### RT-PCR

Total RNA was purified from cell lysates using Trizol reagent (Invitrogen). 2 µg of total RNA was used for reverse transcription using Anchored Oligo-(dT)_23_ (Sigma) as primer for 1st strand synthesis using the RT-AMV kit (Roche). 1:100 dilutions of cDNA were used as template for quantitative PCR using iQ-SYBR Green Master Mix (Biorad) in a Biorad Opticon 2 cycler. Q-RT-PCR values were normalized to *ACTB* mRNA levels for all samples. Primer pairs for RT-PCR of *ACTB, PCNA*, *TFF1*, *MYC*, *STC2*, and *DCC1* are listed in the supplementary materials (Table S1).

### Western Blotting

Cell lysates in 1% SDS lysis buffer were quantified using the Micro BCA Protein Assay Kit (Pierce), and 30 µg of total protein per well was separated by SDS-PAGE, transferred to PVDF membranes, and probed with antibodies against ACTIN (Sigma #A4700, used at 1:500 dilution) or PCNA (Cell Signaling #2586, used at 1:1000 dilution). Secondary Goat anti-mouse IgG antibody (conjugated with horseradish peroxidase) was incubated at a dilution of 1:10,000 and blots were developed using Amersham ECL Plus Western Blotting Detection Reagents (GE Healthcare).

### Chromatin Immunopreciptation (ChIP)-PCR

ChIP was performed as previously described [31]. Briefly, MCF7 Cells were E2-deprived for 3 days (details above) and then treated with 10 nM E2 or vehicle (45 minutes) at 80% confluence. 45 minutes of E2 exposure has been demonstrated to produce maximal ERα binding to chromatin [72], [78]. ∼5×10^6^ cells per ChIP were cross-linked with 1% formaldehyde for 10 minutes at 37°C then quenched with 125 mM glycine. The cells were washed with cold PBS and scraped into PBS with protease inhibitors (Roche). Cell pellets were resuspended in ChIP lysis buffer (1% SDS, 10 mM EDTA, 50 mM Tris-HCl (pH 8.1) and sonicated (Fisher Sonic Dysmembrinator) to produce sheared chromatin with average length 500 bp. The sheared chromatin was submitted to a clarification spin and the supernatant then used for ChIP or reserved as “Input.” Antibodies used were anti-ERα (Ab-1, Ab-3, and AB-10 from Lab Vision and MC-20 from Santa Cruz). Forward and reverse primer sequences used for ChIP-PCR are listed in the supplementary materials (Table S1).

### Luciferase Reporter Assays

Luciferase reporter assays were performed using the Luciferase Assay System (Promega) according to the manufacturer's instructions. Potential regulatory elements were cloned into pGL2-Promoter (Promega) and transfected into MCF7 cells using the TransIT-LT1 Transfection Reagent (Mirus). Cotransfection with a β-galactosidase expressing plasmid (Promega) enabled normalization of transfection efficiency across samples using a β-galactosidase assay kit (Promega) according to the manufacturer's instructions.

### Cloning and Mutagenesis

PCR cloning was performed using PCR amplification of genomic loci from HESC cell genomic DNA which was prepared using the Genomic DNA Extraction kit (Qiagen) according to the manufacturer's instructions. PCR products were ligated with the reporter construct pGL2-promoter (at 5′-KpnI+3′-XhoI sites) for use in Luciferase Reporter assays (Promega). Mutagenized reporter constructs were prepared using the Genetailor Site-Directed Mutagenesis System (Invitrogen) according to the manufacturer's instructions. All clones and subclones were confirmed by DNA sequencing. Primers used for genomic locus amplification and for subcloning are listed in the supplementary materials (Table S1).

### Statistics

Comparisons between two groups were made using a two-tailed Student's t-test with P values indicated.

## Supporting Information

Table S1 Table of oligonucleotide primers used for cloning, mutagenesis, ChIP-PCR, RT-PCR, and EMSA.
(0.04 MB XLS)

Table S2 Estrogen-responsive transcription factors with predicted binding sites at the 5′- and/or 3′-promoter regions for the PCNA gene.
(0.06 MB XLS)
